# Supplementary material for: The presence of ancient subducted oceanic crust contributes to seismic anomalies in Large Low Shear Velocity Provinces
Source: Commun Earth Environ. 2025 Aug 28;6(1):713. doi: 10.1038/s43247-025-02700-8 (PMC12394072; doi:10.1038/s43247-025-02700-8)
Supplement: Supplementary file 2 — Supplementary Information [file 43247_2025_2700_MOESM2_ESM.pdf]

Supplementary Information

The presence of ancient subducted oceanic crust contributes to seismic anomalies in Large Low Shear Velocity Provinces

Ewa Krymarys[1]*, Motohiko Murakami[1]*, Pinku Saha[1], Christian Liebske[1]

Corresponding author: ewa.krymarys@eaps.ethz.ch, motohiko.murakami@eaps.ethz.ch

The PDF file includes:

Materials and Methods
Supplementary Text (1-5)
Figs. S1 to S11
Tables S1 to S9
Supplementary References

Materials and Methods

Starting Materials

Polycrystalline samples of $CaCl_2$-type SiO_2 and α-PbO_2-type SiO_2 phases were used as starting materials. $CaCl_2$-type SiO_2 phase was obtained from cold compression of stishovite, that was pre-synthesized in a large volume press at 11 GPa and 1200°C. α-PbO_2-type SiO_2 phase was obtained from α-cristobalite, synthesized from SiO_2 fine powder at high temperature (~1400°C). FTIR spectroscopy was conducted at ETH, Zurich using a Bruker Tensor II FTIR attached to a Bruker Lumos II microscope[1–3]. FTIR spectra were acquired using a liquid nitrogen cooled Mercury Cadmium Telluride (MCT) detector in a standard transmission FTIR spectroscopy (mid IR range). The wavenumber resolution of 4 cm^{-1} and 32 seconds of transmission time were adopted in the measurement. The detection limit of H_2O in such a setup was shown to be around 400 ppm in apatite[2]. Total water bands were obtained through a linear baseline generation between wavenumbers 2700 cm^{-1} and 3700 cm^{-1}, for which the linear absorbance of the H_2O band was centered at ~3500 cm^{-1}. It resulted in a total water content below the detection limit of 0.04 wt.% for both stishovite and α-cristobalite starting materials.

Brillouin and Raman Scattering Measurements

Both Brillouin and Raman scattering spectroscopic measurements were performed at ETH Zürich at room temperature in a symmetric Diamond Anvil Cell (DAC) with a 60° angular opening, utilizing a 532 nm wavelength solid state laser. The inelastically scattered photons in two methods were collected in two different geometries: for Raman measurements, it was in a backscattering direction, while in Brillouin measurements, it was in a 50° forward scattering direction. For Brillouin spectra, the scattered light from the center of the sample was collected, passed through a Sandercock-type six-pass Fabry-Perót interferometer, and recorded with a multichannel analyzer[4]. The scattered light contains an elastically scattered photon with frequency w and inelastically scattered photon generated from the interaction between the laser probe and thermally generated photons in the sample with a frequency shift Δw. In a symmetric scattering geometry, the sound velocity V can be calculated without any prior knowledge of the sample refractive index following the relation[5];

$$V_i = \frac{\Delta w_i * \lambda}{2Sin(\theta/2)} \qquad (1)$$

Where λ is the laser wavelength (532 nm), θ is the external scattering angle, and i denotes one of the acoustic modes. To ensure the scattering angle at 50°, BK7 was used as a calibrant. At each pressure point, the sound velocity measurements were carried out at ~3-4 rotational angles of the DAC, and their average value is reported. The typical pressure increments were 4-6 GPa. The collected raw Brillouin spectra of Stokes and anti-Stokes peaks were fitted with a Gaussian peak function to calculate the exact peak position. The uncertainties derived from the peak fitting were quite small (within the range of ~ ±0.02-0.03) with respect to the uncertainties derived from the difference in *Vs* values between Stokes and anti-Stokes Brillouin peaks (within the range of 0.01-0.2 km/s, up to ~1.8% difference), that were averaged out for the final *Vs* determination. The collection time of Brillouin spectra varied from 2 h up to 3 days, depending on the sample and pressure conditions. Shorter acquisition times were adopted in α-PbO_2-type SiO_2 phase and much longer times in $CaCl_2$-type SiO_2 sample. Raman signals were dispersed using 2400gr/mm grating with a typical acquisition time of 10-30 minutes in each spectrum range. Raman spectroscopic measurements allowed us to determine the pressure condition before and after each Brillouin measurement through the high-frequency edge shift of the diamond (T_{2g} mode)[6–8] the phase

identification (Fig. S5). The pressure distribution within the centrally-measured areas of samples (~20 μm in size) varied within 1-2 GPa (Table S1), where the spot size of both Raman and Brillouin measurements were ~5-10 μm.

Sample configuration in Diamond Anvil Cells

All samples were pressed to pellets and loaded into the sample chamber in a diamond anvil cell with a thickness of ~30 μm. The polycrystalline stishovite and α-cristobalite were compressed in rhenium gaskets, serving as sample chambers with pre-indented thickness of ~40 μm and a hole diameter of ~55-65 μm. A very thin layer of alkali halide (NaCl) was applied on both sides of the samples, acting as a pressure medium.

Synchrotron X-ray Diffraction Measurements

The structure of all examined samples was checked at European Synchrotron Radiation Facility (ESRF) in Grenoble, France, at high pressure (90-100 GPa). The X-ray beam was focused to ~6 μm, both vertically and horizontally, with a fixed wavelength at 0.3738 Å (33.17 keV). The detector to sample distance, beam center, and detector tilt were calibrated using a CeO_2 standard.

A more detailed description of the steps undertaken and analyses in this study can be found in the Supplementary Text (1-5).

Supplementary Text (1-5)

1) Structure Determination

Based on the obtained XRD patterns, we performed a structure analysis with the use of Dioptas[9] and PDIndexer program[10], by adopting the K_0, K_0', V_0 and lattice parameters from[11–13] for $CaCl_2$-type SiO_2 phase and [11,13–15]for α-PbO_2-type SiO_2 phase. The adopted site occupancies and space group of the $CaCl_2$-type phase were taken from the PDIndexer depository, whereas the α-PbO_2-type was considered in a threefold way. First, as a *Pnc2* space group in case of fitting our data to[14], second as monoclinic $P2_1/n$ space group in case of fitting our data to cristobalite X-I of[15], and third as orthorhombic *Pbcn* space group in case of fitting our data to [11,13]. In the latter case, the theoretical space group of α-PbO_2-type[16] was adopted from the online WURM depository. As a result, we obtained the best *d*-spacing values in all our samples with the study of Grocholski et al., 2013[13] (Fig. S3-S4), with the error range within ~0.2-0.4%. The highest error in X-ray analyses in our study was found when compared to a monoclinic polymorph of silica, cristobalite X-I[15]. This could be explained because of the lower pressure polymorph, cristobalite X-I[15], that was found similar to the higher-pressure polymorph of α-PbO_2-type SiO_2 phase (seifertite), upon decompression at 50 GPa[13,16–18].

The high-pressure and high-temperature phase relations of silica with respect to a starting material and synthesis conditions are complex, as indicated by[13,14,19]. Even though multiple metastable high-pressure silica polymorphs might form, depending on the pressure-temperature conditions, the recalculation of several metastable silica polymorphs (α-PbO_2 (seifertite), postquartz (*3 x 2* type or $P2_1/c$ type), $NaTiF_4$ type and SnO_2 type)[13] showed that the structural differences among them are minor. This implies that the α-PbO_2-type most probably belongs to the *Pbcn* space group. Earlier studies of[14,17,20] suggested a lower symmetry of *Pnc2*[14] because it was required to perform a good fit. Hence, the monoclinic distortions in α-PbO_2-type *Pbcn*[17] and in the preceding lower silica polymorph of cristobalite X-I[15] were considered. This can be possible, but since this is difficult to discern with X-ray studies, the inclination towards the *Pbcn*

space group in α-PbO_2-type SiO_2 phase (seifertite) has been postulated as plausible in α-cristobalite[17,20] and followed by other researchers in SiO_2 system[11,13,21,22]. For the sake of simplicity, we are referring to the *Pbcn* space group with theoretically established symmetry information derived from the WURM online depository[16].

We performed Raman measurements, as a complementary technique for all our examined materials and found a good match with[23] for our $CaCl_2$-type SiO_2 phase, and with[15,17] for our α-PbO_2-type SiO_2 phase. The Raman spectra can be seen in Fig. S5 with intensified A_{1g} mode and reduced B_{1g} intensity mode, that is characteristic of $CaCl_2$-type stability field. Similarly to[23], we found an unwanted peak at ~550 cm^{-1} in our $CaCl_2$-type SiO_2 phase, that was detected, however at ~475 cm^{-1} in the previous study[23]. Since previous study[23] did not investigate the pressure evolution, we can observe that in our study, this feature is fixed, irrespective of the pressure condition. This would imply a possible unwanted scattering coming from the Raman system. In the α-PbO_2-type structure, four A_{1g} and three B_{2g} modes were detected, confirming the structure with previous studies[15,17].

2) Pressures Determined Using XRD Data

The PDIndexer program was used to determine the actual volume and pressure conditions in our samples by following the equation of state (EoS) of the examined phases (Fig. S10-S11).

3) Finite Strain Fitting

We calculated P-V_S profiles of all examined materials and subsequently fitted them to the third-order Birch-Murnaghan equation, that is an isothermal Eulerian finite-strain (*f*) EoS correlating pressure and volume with the use of the BurnMan toolkit[24,25] (Fig. 2, Table S2). The method applied the isothermal shear modulus equation, derived from[24]:

$$G = (1 + 2f)^{5/2}[G_0 + (3*K_0*G_0' – 5*G_0)*f + (6*K_0+G_0' – 24*K_0 – 14*G_0 + 9/2*K_0*K_0')*f^2]$$

(1) that allowed us to determine the adiabatic shear modulus (G_0) and its pressure derivative ($G_0' = dG/dP$). In order to perform the fitting, the input data of volume at 0 GPa (V_0), the isothermal bulk modulus (K_0), and its pressure derivative (K_0') were adopted from the previous study[13], that gave us the best X-ray fit to all our examined materials. Fig. 2 represents our G_0 and G_0' fits with[13] for all examined samples and compares them with the only existing study on acoustic wave velocities in polycrystalline stishovite with its $CaCl_2$-type phase[26] up to ~60 GPa.

4) Temperature Calculation in Modeling

The temperature was calculated in our modeling with the use of BurnMan third-order Birch-Murnaghan fit, supplemented with the Mie-Grüneisen EoS, as specified in the study of[24]. The thermodynamic parameters of θ, γ, q, η were derived from the available library, coming from theoretical study of[27] (slb_2011 that are based on third-order fit slb3 EoS).

To explain the formulas and correlations, we provide information coming from the study of[24]. Thermal correction was adopted on pressure (*P*) and shear modulus (*G*), following the Mie-Grüneisen-Debye EoS with the quasi-harmonic approximation, as below:

$$P_{th}(V, T)=\frac{\gamma \Delta U}{V}, (1)$$

$$G_{th}(V, T)=-\frac{\eta_S \Delta U}{V}, (2),$$

Where: Δ is a difference in the relevant quantity from the reference temperature (300 K), γ is the Grüneisen parameter, η_s is the shear strain derivative of the Grüneisen parameter, and U is the internal energy at temperature T.

In our modeling, we examined MORB, which is a multiphase component (assemblage), with the phase proportions specified in Table S3. The constituent phases of MORB followed the Voigt-Reuss-Hill average bound schemes (eq.3), for which the resultant V_S profile was calculated, following a cold slab geotherm or lower mantle geotherm[28], as presented in Fig.S8.

$$X_{VRH}=\frac{1}{2}(X_V + X_R), (3)$$

5) Estimation of Stress Conditions

The estimation of stress was performed based on the X-ray diffraction data obtained under high pressure conditions, corresponding to 92 and 99 GPa for $CaCl_2$-type and α-PbO_2-type, subsequently. The non-hydrostaticity in examined samples can be characterized by the systematic deviation of the measured lattice parameters from the hydrostatic value depending on the *hkl* indices[29].

Therefore, the uniaxial stress component t in cubic system can be defined by the lattice parameter a_m (*hkl*), the orientation of diffracting plane, and the elastic modulus of the sample as follows:

$$a_m (hkl) = M_0 + M_1 [3(1-3\sin^2\theta)\Gamma (hkl)], (1)$$

where:

$$M_0 = a_p \{1 + (\alpha t/3)(1-3\sin^2\theta) [S_{11} - S_{12} - (1-\alpha^{-1})/(2G_v)], (2)$$

$$M_1 = -a_p \alpha t S/3, (3)$$

$$\Gamma (hkl) = (h^2k^2 + k^2l^2 + l^2h^2)/(h^2 + k^2 + l^2)^2, (4)$$

$$S = S_{11} - S_{12} - S_{44}/2, (5)$$

Here a_p is the lattice parameter under hydrostatic pressure, θ denotes the diffraction angle. The α decides the actual stress of the sample that takes value between 0.5 and 1. G_V is the shear modulus under isostrain condition. S_{ij} is the single-crystal elastic compliance. If $M_0 \sim a_p$ is assumed in equation (3), the uniaxial component t can be expressed as the following relation:

$$t \sim -3M_1/(\alpha M_0 S), (6)$$

The $M1$ and $M0$ correspond to the slope and intercept of a plot $a_m (hkl)$ versus $3(1-3\sin 2\ \theta)\Gamma$ (*hkl*), called Γ plot, and t can be eventually obtained from the results of $M0$ and $M1$ together with α and S. In accordance with the previous studies[30–34], we also adopted the value $\alpha = 1$. The elastic anisotropy factor S in cubic system can be expressed by Cij as:

$$S = 1/(C_{11} - C_{12})-1/(2C_{44}), (7)$$

In order to assess the stress conditions in our samples we plotted Γ plots for all examined samples (Fig.S1-S2).

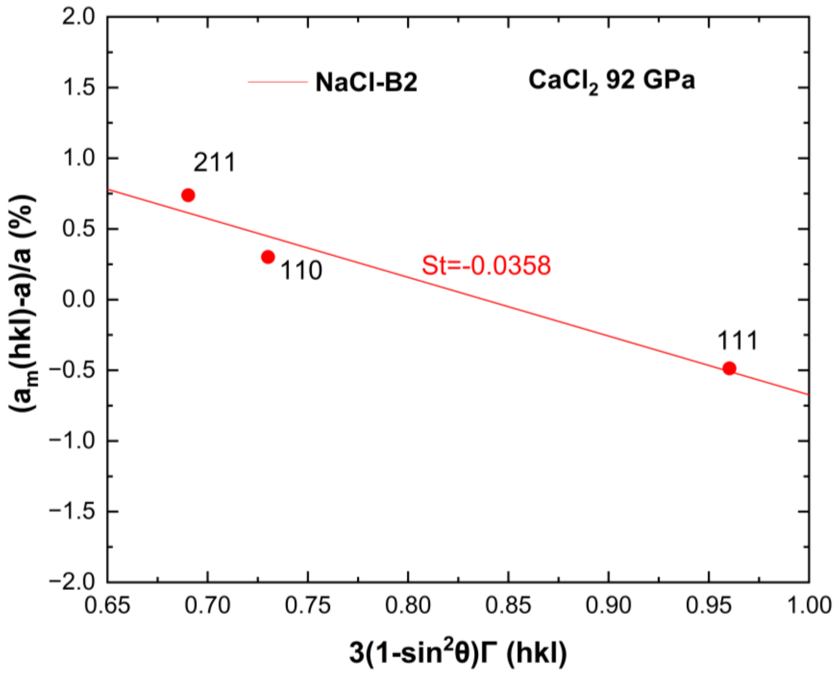

Fig. S1. The Γ plot for SiO_2 $CaCl_2$-type phase.

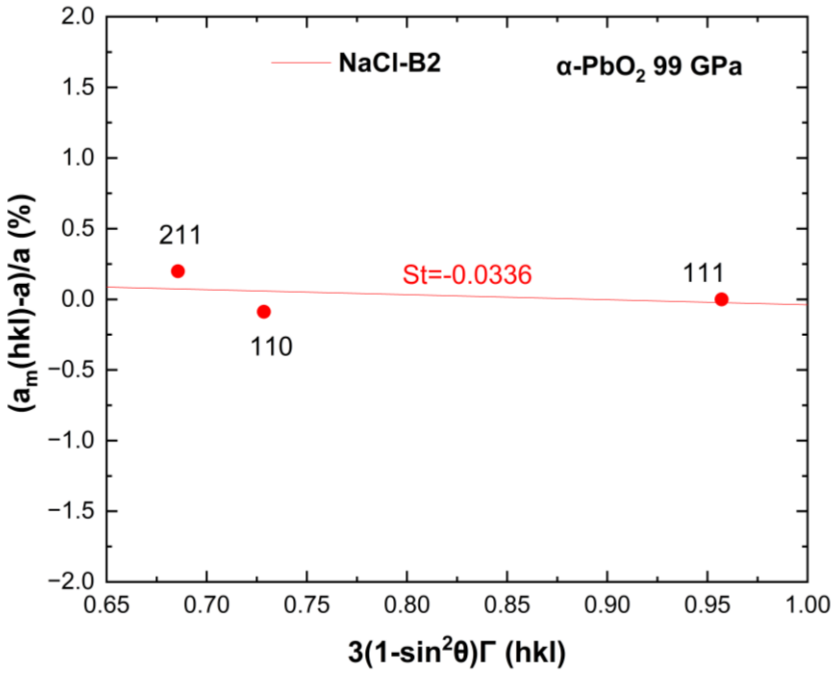

Fig. S2. The Γ plot for SiO_2 α-PbO_2-type phase.

By applying the available pressure dependence of *S* values in NaCl[35], the stress was estimated to be ~5 GPa and ~6 GPa for the $CaCl_2$ and α-PbO_2 SiO_2 phases, respectively.

In addition to this, we examined how a pressure change of ~5 GPa and ~6 GPa at pressure of ~100 GPa affects V_S using the obtained shear wave velocity profile from finite strain fitting. As a result, we found that, under pressure at 100 GPa, the effect on V_S in $CaCl_2$ sample is found to be 0.46% (± 0.035 km/s in V_S), while the effect on V_S in α-PbO_2 sample 0.68% (±0.05 km/s), which are below the experimental errors of V_S we determined (Table S1).

Since all our measured samples were fully surrounded by the pressure medium (NaCl-B2), the stress conditions indicated from the pressure media should most likely represent the same stress conditions within sample chamber. According to the previous literature regarding the stress estimation in a DAC under pressure, it has been very common to use a small piece of Pt as the stress gauge within the sample, e.g.[30]. Comparison of stress conditions between such conventional configurations of (Pt), examined in KCl pressure medium before and after annealing in the study of[36], as well as the in the pressure medium of NaCl[29], imply that the stress of the sample is suppressed following both NaCl-B2[29] or Pt[36] pressure scales. Therefore, the stress conditions of the samples, which are surrounded by the pressure medium and measured at the central locations, would be well suppressed, almost equivalently to the pressure medium or even lower.

By following the study of[37], the differential stress in $CaCl_2$-type sample can be estimated from the shear modulus and the average *Q hkl* value from all measured reflections, following equation (8):

$$t = 6G\{Q(hkl)\}, (8)$$

The estimated stress calculation within the sample of $CaCl_2$-type from the pressure condition measured at synchrotron at 92 GPa equals to t = 2.03 GPa in $CaCl_2$-type, which is slightly below the value of estimated stress condition in NaCl-B2 pressure medium t ~5 GPa. This agrees well with the consensus on pressure medium effect that fully surrounds sample in the central location, where stress condition is equivalent to the one in pressure medium or it is slighlty lower.

Another way to indirectly assess the stress conditions in our samples is based on a correlation between the hydrostaticity within the sample and the pressure distribution/gradient over the sample[38–40]. This implies that the non-hydrostaticity can be considered to be well suppressed if the pressure gradient over the DAC sample is reasonably small. The pressure distribution within the sample area (~20 µm in size) where we performed all the measurements including Brillouin, Raman, and X-ray falls within the range of 1-2 GPa variation within a single pressure point consisting of several angles (Table S1, Supplementary Text, 2), which is reasonably small. This observation would be also indirect supportive evidence that the non-hydrostaticity in our DAC samples remained well suppressed.

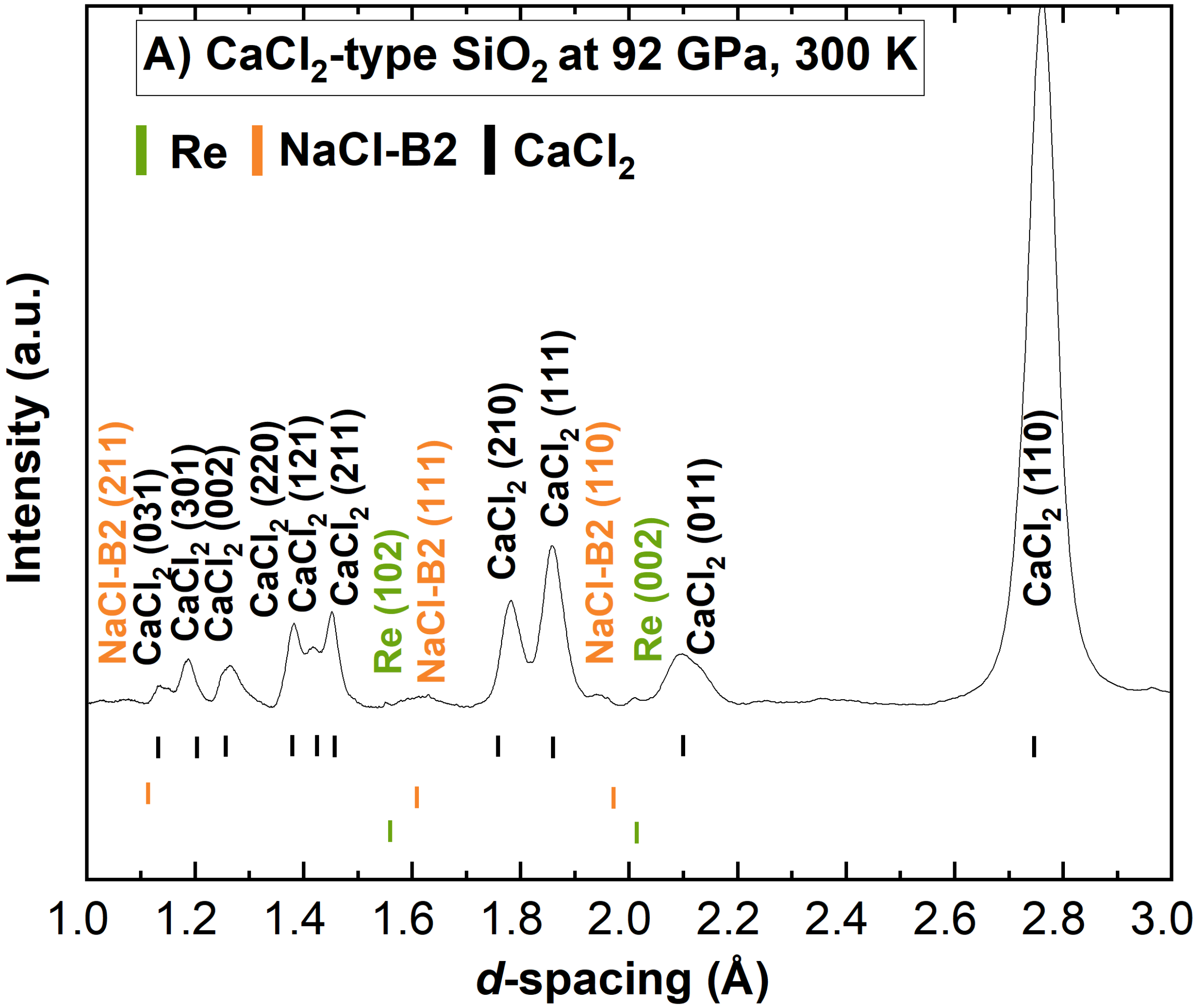

Fig. S3. X-Ray diffraction profile of the examined $CaCl_2$-type SiO_2 phase (derived from polycrystalline stishovite) at 92 GPa and 300 K. Calculated peak positions of $CaCl_2$-type and NaCl-B2 phases as well as Rhenium are marked with their corresponding colors. We found the best *d*-spacing fit with the study of[13] (see more details in Supplementary Text 1-2).

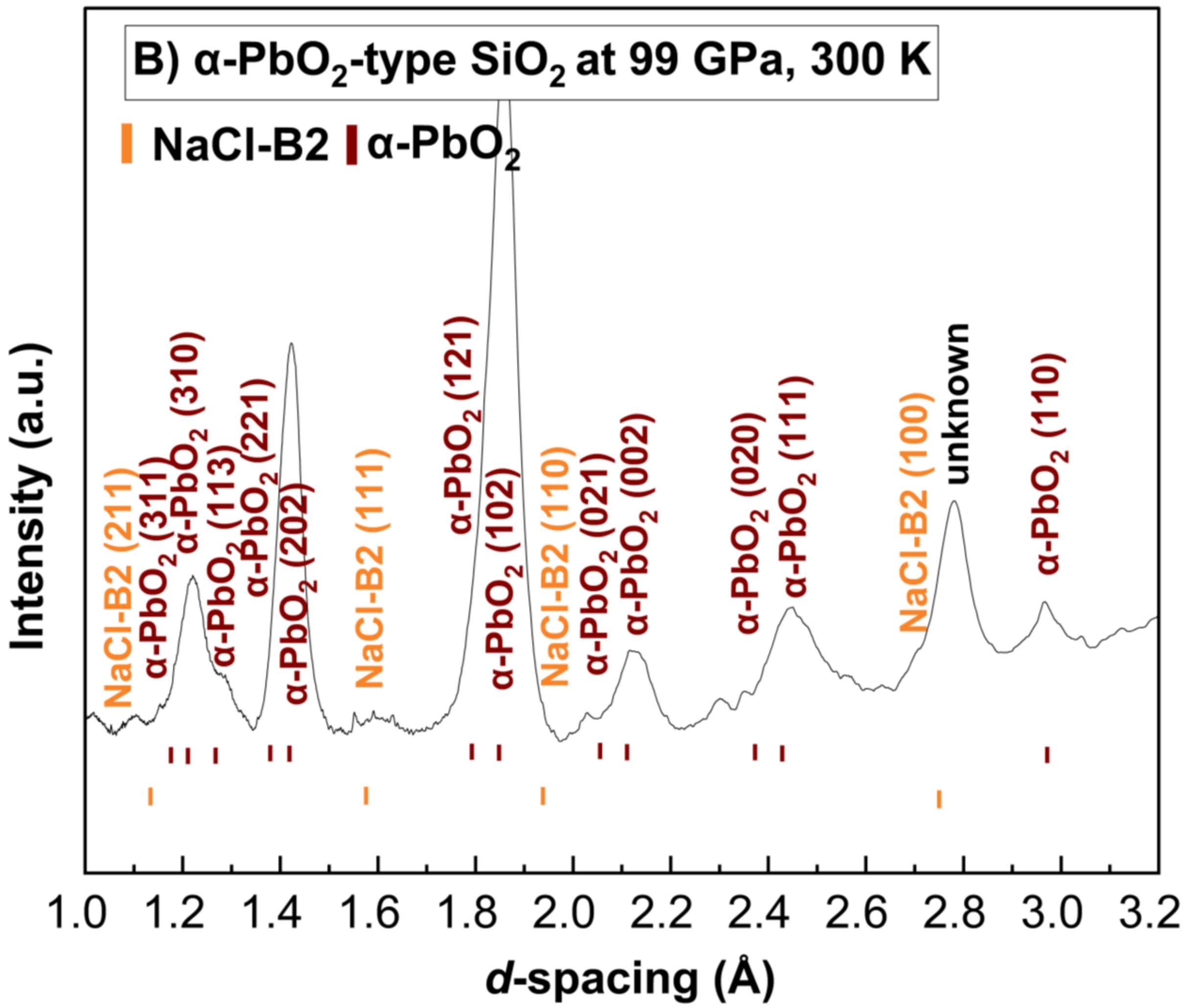

Fig. S4. X-Ray diffraction profile of the examined α-PbO_2-type SiO_2 phase (derived from polycrystalline α-cristobalite) at 99 GPa and 300 K. Calculated peak positions of α-PbO_2-type, NaCl-B2 phases as well as Rhenium are marked with their corresponding colors. We found the best *d*-spacing fit with the study of[13] (see more details in Supplementary Text 1-2).

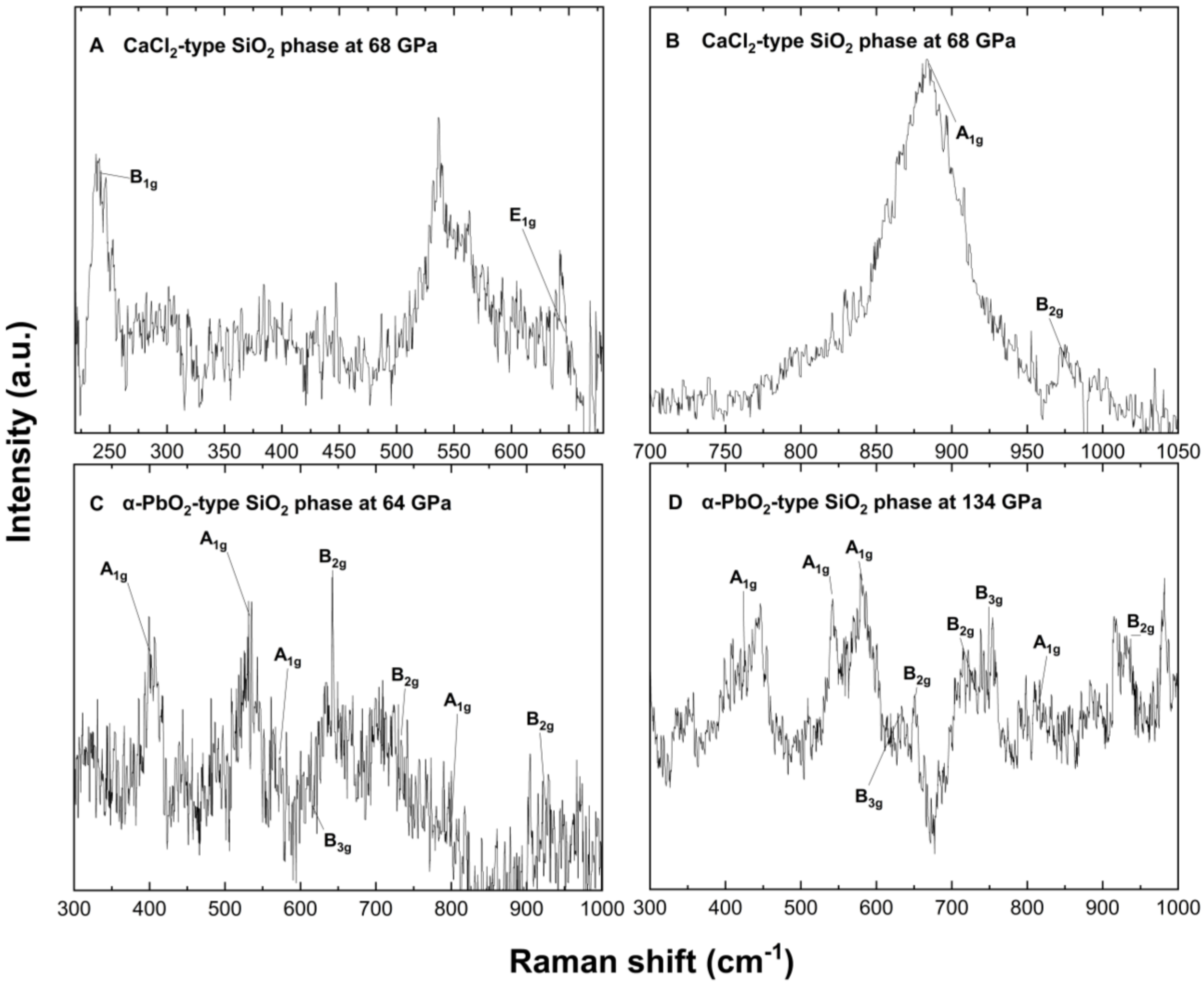

Fig. S5. Raman spectra collected upon compression of all examined samples. A), B), $CaCl_2$-type SiO_2 phase at 68 GPa; C), D), α-PbO_2-type phase (space group *Pbcn*) at 64 and 134 GPa, respectively.

Table S1. Fitted values of acoustic wave velocities using gaussian function to the Brillouin peaks of the polycrystalline $CaCl_2$-type SiO_2 and α-PbO_2-type SiO_2 phases at different pressures (P) with their errors and angular dispersion on V_S.

	Runs	Pressure (GPa)	V_S (km/s)	number of runs	angular dispersion on V_S (%)
**$CaCl_2$-type SiO_2 phase**	P0 average	57(1)	7.20(8)	3	1.2
	P1 average	61.0(4)	7.27(6)	6	0.8
	P2 average	67.3(7)	7.29(4)	4	0.6
	P3 average	74(2)	7.33(4)	3	0.6
	P4 average	77.0(2)	7.43(2)	3	0.3
	P5 average	81.2(3)	7.51(6)	4	0.8
	P6 average	85.0(3)	7.52(4)	3	0.5
	P7 average	91.0(9)	7.54(4)	2	0.5
	P8 average	96.0(3)	7.58(4)	3	0.5
	P9 average	101.0(5)	7.63(8)	2	1.1
	P10 average	105.2(7)	7.65(4)	2	0.6
	P11 average	111(2)	7.65(0)	2	0.0
	P12 average	116.0(2)	7.69(8)	2	1.0
	P13 average	120.0(5)	7.71(5)	2	0.7
	P14 average	126.0(7)	7.77(3)	2	0.4
	P15 average	130.0(7)	7.78(7)	2	0.9
**α-PbO_2-type SiO_2 phase**	P0 average	43.3(1)	6.56(4)	7	0.6
	P1 average	57.2(4)	6.85(3)	5	0.5
	P2 average	63.0(3)	6.93(2)	5	0.3
	P3 average	72(1)	7.06(4)	7	0.6
	P4 average	80.0(7)	7.19(4)	5	0.6
	P5 average	86(1)	7.25(5)	5	0.7
	P6 average	93(2)	7.34(7)	5	1.0
	P7 average	99(2)	7.37(5)	7	0.7
	P8 average	103.0(8)	7.43(4)	5	0.5
	P9 average	108(2)	7.52(3)	5	0.4
	P10 average	114.3(8)	7.56(3)	5	0.4
	P11 average	120.0(9)	7.63(4)	4	0.6
	P12 average	124.0(2)	7.68(4)	3	0.6
	P13 average	128.0(5)	7.7(6)	3	0.7
	P14 average	133.0(7)	7.72(5)	5	0.6
	P15 average	137.2(7)	7.76(4)	5	0.6
	P16 average	143.0(4)	7.8(5)	4	0.6
	P17 average	148.1(5)	7.81(2)	5	0.3

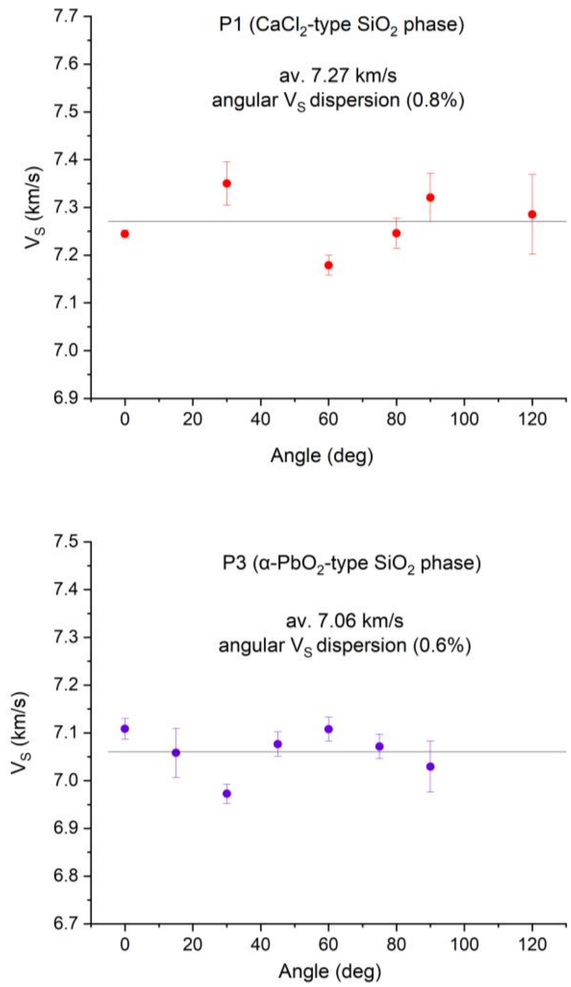

Fig. S6. Angular dispersion of V_S for the $CaCl_2$-type SiO_2 phase at P1 (61 GPa) and the α-PbO_2-type SiO_2 phase at P3 (72 GPa). Error bars represent uncertainties in V_S measurements at each angle.

Table S2. Calculated shear moduli (G_0), and pressure derivatives (G_0') of all SiO_2 phases at 0 GPa, following the volumes (V_0), bulk moduli (K_0) and pressure derivatives (K_0') from the best-fitted study[13]. The obtained lattice constants *a, b, c*, densities (ρ) and volumes (*V*) for $CaCl_2$-type and α-PbO_2-type e SiO_2 phases at 92 and 99 GPa conditions, respectively are compared to[13].

Parameter	**$CaCl_2$-type SiO_2 phase**	**α-PbO_2-type SiO_2 phase**
Grocholski et al., 2013[13]		
Pressure up to (GPa)	152	
V_0 (Å^3)	46.63	91.66
K_0 (GPa)	317	322
K_0'	4	4
Pressure Medium	Argon, Neon	
ρ_0 (g/cm^3)	4.28	4.35
Our results		
Parameter	**$CaCl_2$-type SiO_2 phase (92 GPa)**	**α-PbO_2-type SiO_2 phase (99 GPa)**
G_0 (GPa)	180 (±2)	148 (±2)
G_0'	1.56 (±0.02)	1.67 (±0.01)
ρ (g/cm^3)	4.30 (±0.02)	4.37 (±0.02)
a (Å)	3.98 (±0.00)	3.82 (±0.02)
b (Å)	3.81 (±0.01)	4.66 (±0.02)
c (Å)	2.52 (±0.00)	4.19 (±0.00)
V (cm^3/mol)	11.54 (±0.02)	11.24 (±0.03)

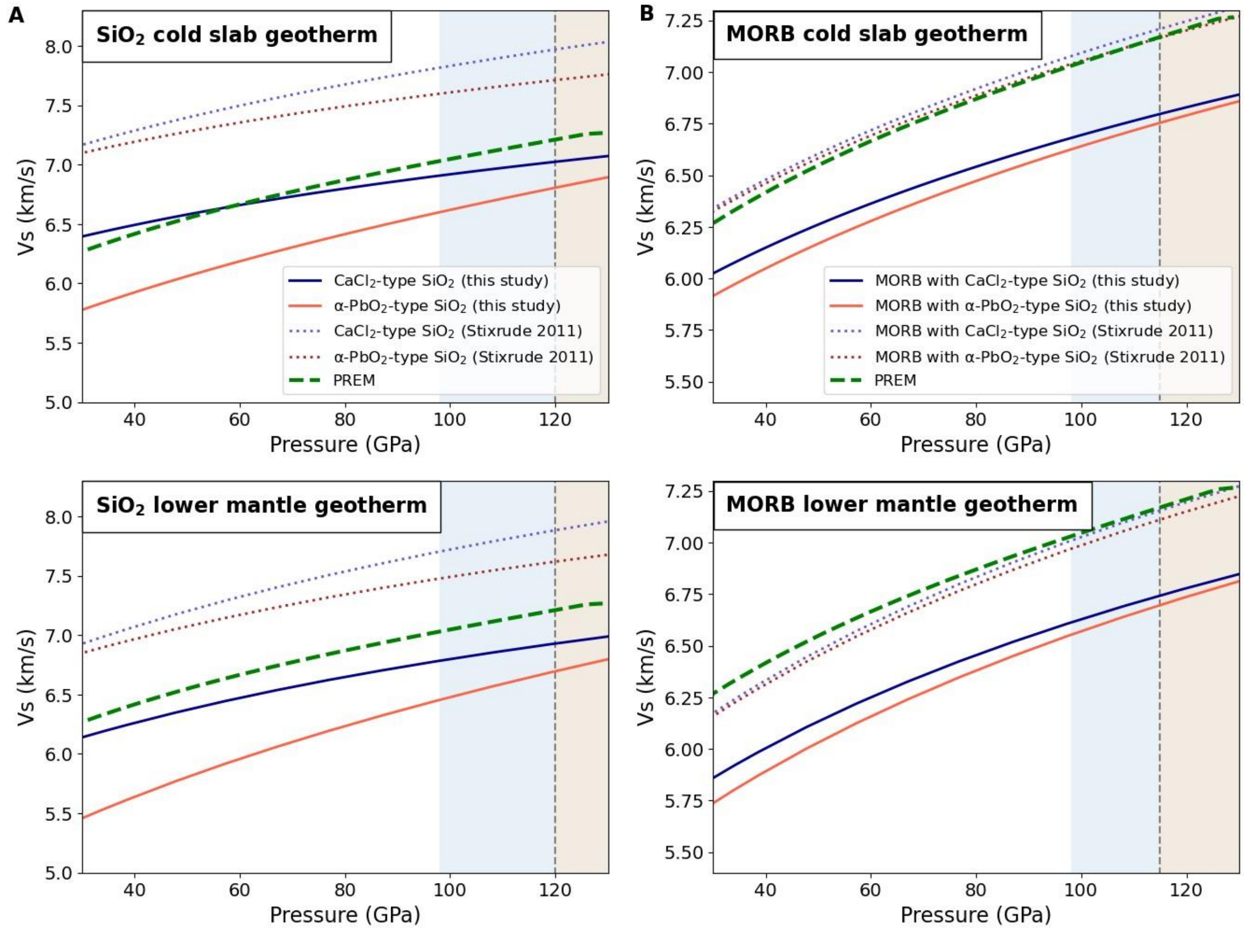

Fig. S7. Comparison between our and computational results[27] on the shear wave velocity profiles of SiO_2 phase (A) and MORB assemblage (B) as a function of pressure along with cold slab-[41] and lower mantle-[28] geotherms (see Tables S4, S6 for difference in theoretically-determined shear wave velocity profiles and contrasts (ΔV_S)). The blue shaded area indicates the expected pressure range where the LLSVPs are primarily observed[42]. The orange area represents the stability field range of α-PbO_2-type SiO_2 phase[11–13,43,44].

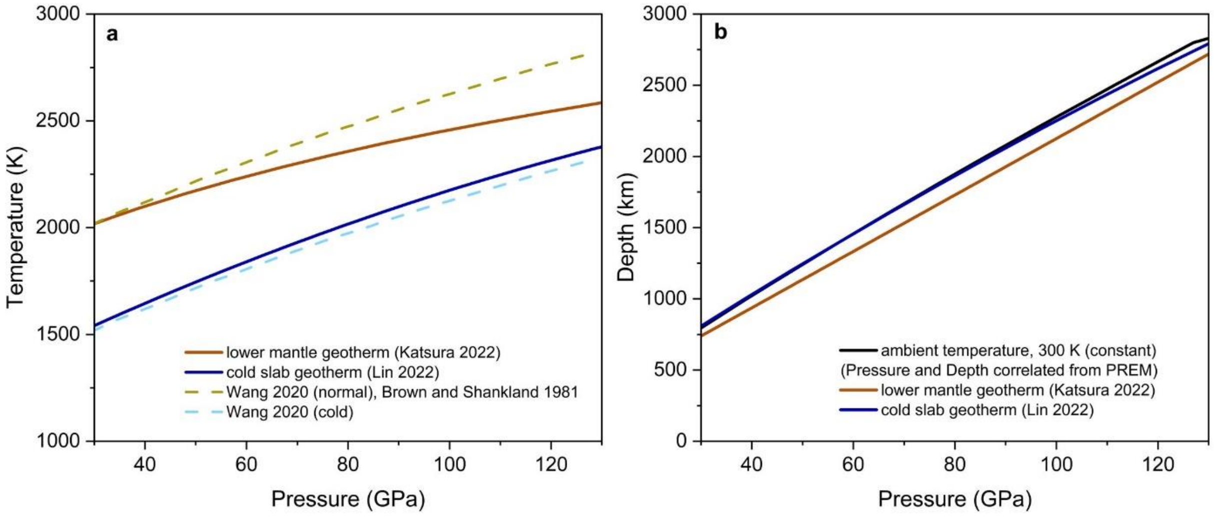

Fig. S8. The adopted temperature profiles in this study: cold slab[41] and lower mantle[28] geotherms a), and the correlation between pressure and depth in the examined temperature profiles b). The geotherms in this study are compared to Brown and Shankland profile[45], adopted as a normal geotherm in theoretical study of[46], and to Brown and Shankland profile[45], reduced by 500 K, and adopted as a cold geotherm in theoretical study of[46].

Table S3. The adopted MORB composition from Hirose et al., 2005[47] with calculated molar fractions, corresponding to 60 and 113 GPa conditions.

MORB at 60 GPa						
wt.% in MORB		molar fraction in MORB	molar fraction in Pv		molar fraction in CF	
SiO_2	17	0.30	Pv endmembers		CF endmembers	
CaPv	23	0.21	MgPv	0.43	MgCF	0.29
MgPv	35	0.37	FePv	0.30	FeCF	0.29
CF	25	0.12	AlPv	0.27	NaCF	0.42
MORB at 113 GPa						
wt.% in MORB		molar fraction in MORB	molar fraction in PPv		molar fraction in CF	
SiO_2	23	0.37	PPv endmembers		CF endmembers	
CaPv	23	0.19	MgPv	0.40	MgCF	0.29
MgPv	38	0.37	FePv	0.36	FeCF	0.29
CF	16	0.07	AlPv	0.24	NaCF	0.42

Table S4. Thermodynamic parameters used for the calculation of the shear wave velocity profiles of SiO_2 system and MORB assemblage in this and theoretical studies, for which MgPv and CF endmembers are derived from theoretical work[27]. Recent experimental studies on Pv bridgmanite endmembers are provided for comparison. The best X-ray fit was found with[13]. Cubic CaPv[48] was used for MORB assemblage. For MORB assemblage (see Table S3). Cubic CaPv[49] was used for comparison and its effect can be seen in (Fig. S9, Table S6).

**Parameter**	**this study***		**(Stixrude et al., 2011)[27]**		**(Greaux et al., 2019)[48]**	**(Thomson et al., 2019)[49]**	**(Stixrude et al., 2011)[27]**						**experimental Pv endmembers**		
	**$CaCl_2$-type SiO_2**	**α-PbO_2-type SiO_2**	**$CaCl_2$-type SiO_2**	**α-PbO_2-type SiO_2**	**CaPv**	**CaPv**	**MgPv**	**FePv**	**AlPv**	**MgCF**	**FeCF**	**NaCF**	**MgPv**	**FePv**	**AlPv**
V_0 (cm^3/mol)	**14.04***	**13.79***	14.02	13.67	27.45˩	27.45↑	24.45	25.49	24.94	36.18	37.26	36.27	24.43ᵀ	24.54''	24.51⌐
K_0 (GPa)	**317***	**322***	314	328	248˩	248↑	251	272	258	211	211	161	257.1=	256ʿ	252≈
K_0'	**4***	**4***	3.75	4.02	4.2˩	3.6↑	4.14	4.14	4.14	4.05	4.05	4.32	3.71=	4ʿ	3.7≈
θ_0 (K)	1107.82×	1140.77×	1107.82	1140.77	795.78×	795.78×	905.94	870.81	886.46	838.63	804.2	812.48	905.94×	870.81×	886.46×
γ_0	1.37×	1.37×	1.37	1.37	1.89×	1.89×	1.57	1.57	1.57	1.31	1.31	0.69	1.57×	1.57×	1.57×
q_0	2.84×	2.84×	2.84	2.84	0.9×	0.9×	1.11	1.11	1.11	1	1	1	1.11×	1.11×	1.11×
G_0 (GPa)	**180**	**148**	220	227	126˩	107↑	173	133	171	130	154	122	169.6ᵀ	165.8^	162.2⌐
G_0'	**1.56**	**1.67**	1.93	1.77	1.61˩	1.66↑	1.69	1.37	1.5	1.76	1.76	2.08	1.73ᵀ	1.76^	1.81⌐
η_{S0}	4.61×	4.97×	4.61	4.97	1.29×	1.29×	2.57	2.29	2.47	2.11	3.03	2.79	2.57×	2.29×	2.47×

***Grocholski et al., 2013; ×Stixrude et al., 2011; ˩Greaux et al., 2019; ↑ Thomson et al., 2019; ᵀ Murakami et al., 2007[50]; = Criniti et al., 2021[51]; ʿDorfman, Duffy 2014[52]; ^Mashino et al., 2020[53]; ⌐Murakami et al., 2012[54]; ≈Jackson et al., 2005[55].**

Table S5. Thermodynamic parameters used for the calculation of the shear wave velocity profiles of SiO_2 system and MORB assemblage, considering other existing studies[12,14,15,44] with[48] used as a cubic CaPv and with[27] used as MgPv, CF endmembers.

**Parameter/references**	**$CaCl_2$-type SiO_2**		**α-PbO_2-type SiO_2**		
	**(Andrault et al., 2003)[12]**	**(Sun et al., 2019)[44]**	**(Cernok et al., 2017)[15]**	**(Dubrovinsky et al., 2001)[14]**	**(Sun et al., 2019)[44]**
V_0 (cm^3/mol)	13.94	14.48	14.54	14.08	13.89
K_0 (GPa)	334	245	230	313	290
K_0'	4.00	4.00	4.00	3.43	4.00
Calculated G_0 and G_0'					
G_0 (GPa)	182	171	136	143	145
G_0'	1.51	1.80	1.91	1.67	1.75

All remaining thermodynamic parameters (θ_0, γ_0, q_0, η_{S0}), adopted from Stixrude et al., 2011 can be found in Table S4.

Table S6. The average negative shear wave velocity contrasts (ΔV_S) with the expected CaCl$_2$-type to α-PbO$_2$-type SiO$_2$ phase transition[11–13,43,44] in pure SiO_2 (120-125 GPa) and MORB (115-125 GPa)[56] systems following the cold slab-[41] and lower mantle-[28] geotherms**.** The table represents results from this study, where the best X-ray fit was found with the study of[13], the differences in MORB (ΔV_S) , once cubic CaPv is implemented from[48,49], and compares it with theoretical study of[27]. For MORB assemblage (see Table S3).

60 GPa MORB composition for both CaCl$_2$-type and α-PbO$_2$-type SiO_2 phases	**this study***		**theory**ˣ		**this study*, CaPv effect**⌐↑	
	SiO_2	MORB (CaPv Greaux et al., 2019)[48]	SiO_2	MORB (CaPv Stixrude et al., 2011)[27]	MORB⌐(Greaux et al., 2019)[48]	MORB↑ (Thomson et al., 2019)[49]
cold slab geotherm (Lin et al., 2022)[41]	3.0(1)	0.58(6)	3.3(0)	0.61(2)	0.58(6)	0.58(6)
lower mantle geotherm (Katsura 2022)[28]	3.2(2)	0.63(6)	3.4(0)	0.64(2)	0.63(6)	0.63(6)
60 GPa MORB composition for CaCl$_2$-type and 113 GPa MORB composition for α-PbO$_2$-type SiO_2 phase	**this study***		**theory**ˣ		**this study*, CaPv effect**⌐↑	
	SiO_2	MORB (CaPv Greaux et al., 2019)[48]	SiO_2	MORB (CaPv Stixrude et al., 2011)[27]	MORB⌐(Greaux et al., 2019)[48]	MORB↑ (Thomson et al., 2019)[49]
cold slab geotherm (Lin et al., 2022)[41]	3.0(1)	1.1(2)	3.3(0)	0.57(9)	1.1(2)	1.1(2)
lower mantle geotherm (Katsura 2022)[28]	3.2(2)	1.2(2)	3.4(0)	0.58(9)	1.2(2)	1.2(2)

*Grocholski et al., 2013; ˣStixrude et al., 2011; ⌐Greaux et al., 2019; ↑Thomson et al., 2019.

Table S7. The average negative shear wave velocity contrasts (ΔV_S) with the expected $CaCl_2$-type to α-PbO_2-type SiO_2 phase transition[11–13,43,44] in pure SiO_2 (120-125 GPa) and MORB (115-125 GPa)[56] systems following the cold slab-[41] and lower mantle-[28] geotherms. The table represents results from this study, where the best X-ray fit was found with the study of[13], and compares the resultant (ΔV_S) with other existing studies. For MORB assemblage (see Table S3). MORB composition for both $CaCl_2$-type and α-PbO_2-type SiO_2 phases was adopted from 60 GPa conditions[47].

**other studies**	**α-PbO_2-type SiO_2 phase (Dubrovinsky et al., 2001)[14]**		**α-PbO_2-type SiO_2 phase (Cernok et al., 2017)[15]**		**α-PbO_2-type SiO_2 phase (Sun et al., 2019)[44]**		**average**	
**$CaCl_2$-type SiO_2 phase (Grocholski et al., 2013)[13]***	**SiO_2**	**MORB (CaPv Greaux et al., 2019)[48]**	**SiO_2**	**MORB (CaPv Greaux et al., 2019)[48]**	**SiO_2**	**MORB (CaPv Greaux et al., 2019)[48]**	**SiO_2**	**MORB (CaPv Greaux et al., 2019)[48]**
cold slab geotherm (Lin et al., 2022)[41]	3.1(1)	0.61(5)	2.8(1)	0.54(5)	2.9(1)	0.57(5)	3.0(1)	0.58(2)
lower mantle geotherm (Katsura 2022)[28]	3.4(2)	0.65(6)	3.0(1)	0.58(5)	3.2(2)	0.62(6)	3.2(1)	0.62(4)
**$CaCl_2$-type SiO_2 phase (Andrault et al., 2003)[12]**								
cold slab geotherm (Lin et al., 2022)[41]	3.1(1)	0.61(5)	2.8(1)	0.54(5)	2.9(1)	0.57(5)	3.0(1)	0.57(2)
lower mantle geotherm (Katsura 2022)[28]	3.4(1)	0.65(6)	3.0(1)	0.58(5)	3.2(1)	0.62(6)	3.2(1)	0.62(4)
**$CaCl_2$-type SiO_2 phase (Sun et al., 2019)[44]**								
cold slab geotherm (Lin et al., 2022)[41]	3.3(1)	0.65(5)	3.0(1)	0.59(5)	3.2(1)	0.61(6)	3.2(1)	0.62(2)
lower mantle geotherm (Katsura 2022)[28]	3.6(2)	0.70(6)	3.3(1)	0.63(6)	3.4(2)	0.66(6)	3.5(1)	0.67(4)

* this study, (ΔV_S) in MORB: 0.58(6) under cold slab geotherm and 0.63(6) under lower mantle geotherm (Table S6)

Table S8. The average negative shear wave velocity contrasts (ΔV_S) with the expected $CaCl_2$-type to α-PbO_2-type SiO_2 phase transition[11–13,43,44] in MORB (115-125 GPa)[56] system (with SiO_2 phase enrichment from 17 wt.% to 23 wt.%) following the cold slab-[41] and lower mantle-[28] geotherms. The table represents results from this study, where the best X-ray fit was found with the study of[13], and compares the resultant (ΔV_S) with other existing studies. For MORB assemblage (see Table S3). MORB composition for $CaCl_2$-type was adopted from 60 GPa conditions, whereas for α-PbO_2-type SiO_2 phase from 113 GPa conditions[47].

**other studies**	**α-PbO_2-type SiO_2 phase (Dubrovinsky et al., 2001)**[14]	**α-PbO_2-type SiO_2 phase (Cernok et al., 2017)**[15]	**α-PbO_2-type SiO_2 phase (Sun et al., 2019)**[44]	**average**
**$CaCl_2$-type SiO_2 phase (Grocholski et al., 2013)**[13]*****	**MORB (CaPv Greaux et al., 2019)**[48]	**MORB (CaPv Greaux et al., 2019)**[48]	**MORB (CaPv Greaux et al., 2019)**[48]	**MORB (CaPv Greaux et al., 2019)**[48]
cold slab geotherm (Lin et al., 2022)[41]	1.2(2)	1.1(2)	1.1(2)	1.1(0)
lower mantle geotherm (Katsura 2022)[28]	1.2(2)	1.1(2)	1.2(2)	1.2(0)
**$CaCl_2$-type SiO_2 phase (Andrault et al., 2003)**[12]				
cold slab geotherm (Lin et al., 2022)[41]	1.2(2)	1.1(2)	1.1(2)	1.1(0)
lower mantle geotherm (Katsura 2022)[28]	1.2(2)	1.1(2)	1.2(2)	1.2(0)
**$CaCl_2$-type SiO_2 phase (Sun et al., 2019)**[44]				
cold slab geotherm (Lin et al., 2022)[41]	1.2(2)	1.1(2)	1.2(2)	1.2(0)
lower mantle geotherm (Katsura 2022)[28]	1.3(2)	1.2(2)	1.2(2)	1.2(0)

* this study, (ΔV_S) in MORB: 1.1(2) under cold slab geotherm and 1.2(2) under lower mantle geotherm (Table S6)

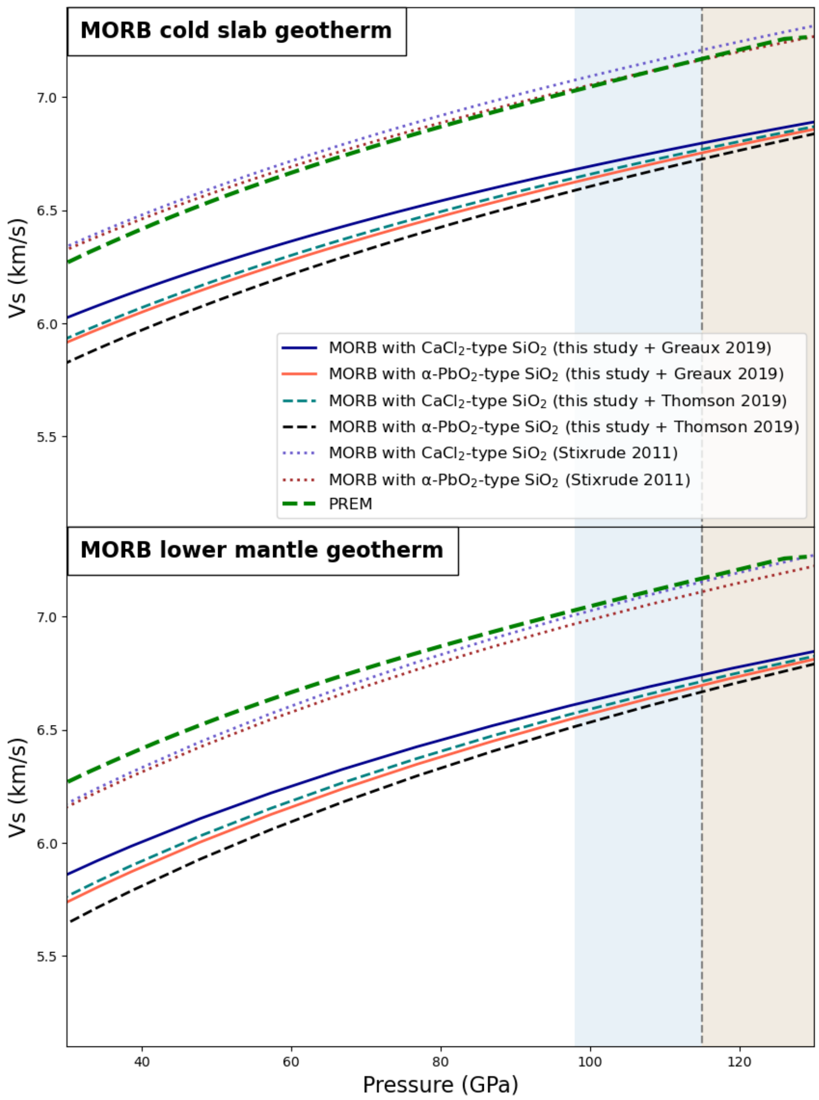

Fig. S9. Shear wave velocity profiles of MORB assemblage as a function of pressure along with the cold slab-[41] and lower mantle[28] geotherms. The adopted MORB composition and its molar fractions can be found in (Table S3). For CaPv phase, recent experimentally-derived cubic phases from[48] and[49] were adopted. The effect of experimentally-derived cubic CaPv[48,49] and theoretically-derived phases[27] in MORB and pure SiO_2 systems can be found in (Tables S4, S6-S8). The blue shaded area indicates the expected pressure range where the LLSVPs are primarily observed[42]. The orange area represents the stability field range of α-PbO_2-type SiO_2 phase[11–13,43,44].

Table S9. The required volume fraction of MORB to explain the seismically observed anomalies in LLSVPs at the CMB ranging from 3000 K to 4000 K, via modeled negative shear wave velocity contrasts (ΔV_S) of -1.5% and -3%. The two potential scenarios, A & B, have been considered: (A) without assuming a change in the volume fraction of SiO_2 due to the phase transition from the $CaCl_2$-type to the α-PbO_2-type structure, (B) assuming a change in the volume fraction of SiO_2 due to the phase transition from the $CaCl_2$-type to the α-PbO_2-type structure, following the MORB's phase proportions from 60 and 113 GPa conditions, respectively[47]. Case 1 follows bridgmanite endmembers from Stixrude et al., 2011[27], whereas case 2 follows bridgmanite endmembers from Murakami et al., 2007[50] and Criniti et al., 2021[51] (MgPv); Murakami et al., 2012[54] and Jackson et al., 2005[55] (AlPv) and Mashino et al., 2020[53] (FePv).

**Case 1** Required volume fraction of MORB in case (A)								
ΔV_S	cold slab geotherm	lower mantle geotherm	3000 K	3800 K	3850 K	3890 K	3900 K	4000 K
-1.50%	23 vol.%	21 vol.%	17 vol.%	12 vol.%	12 vol.%	12 vol.%	12 vol.%	12 vol.%
-3.00%	47 vol.%	42 vol.%	34 vol.%	25 vol.%	24 vol.%	24 vol.%	24 vol.%	23 vol.%
Required volume fraction of MORB in case (B)								
ΔV_S	cold slab geotherm	lower mantle geotherm	3000 K	3800 K	3850 K	3890 K	3900 K	4000 K
-1.50%	22 vol.%	20 vol.%	16 vol.%	12 vol.%	12 vol.%	11 vol.%	11 vol.%	11 vol.%
-3.00%	44 vol.%	39 vol.%	32 vol.%	24 vol.%	23 vol.%	23 vol.%	23 vol.%	22 vol.%
**Case 2** Required volume fraction of MORB in case (A)								
ΔV_S	cold slab geotherm	lower mantle geotherm	3000 K	3800 K	3850 K	3890 K	3900 K	4000 K
-1.50%	33 vol.%	28 vol.%	22 vol.%	15 vol.%	14 vol.%	14 vol.%	14 vol.%	14 vol.%
-3.00%	66 vol.%	56 vol.%	44 vol.%	30 vol.%	29 vol.%	28 vol.%	28 vol.%	27 vol.%
Required volume fraction of MORB in case (B)								
ΔV_S	cold slab geotherm	lower mantle geotherm	3000 K	3800 K	3850 K	3890 K	3900 K	4000 K
-1.50%	32 vol.%	27 vol.%	16 vol.%	12 vol.%	12 vol.%	11 vol.%	11 vol.%	11 vol.%
-3.00%	64 vol.%	54 vol.%	32 vol.%	24 vol.%	23 vol.%	23 vol.%	23 vol.%	22 vol.%

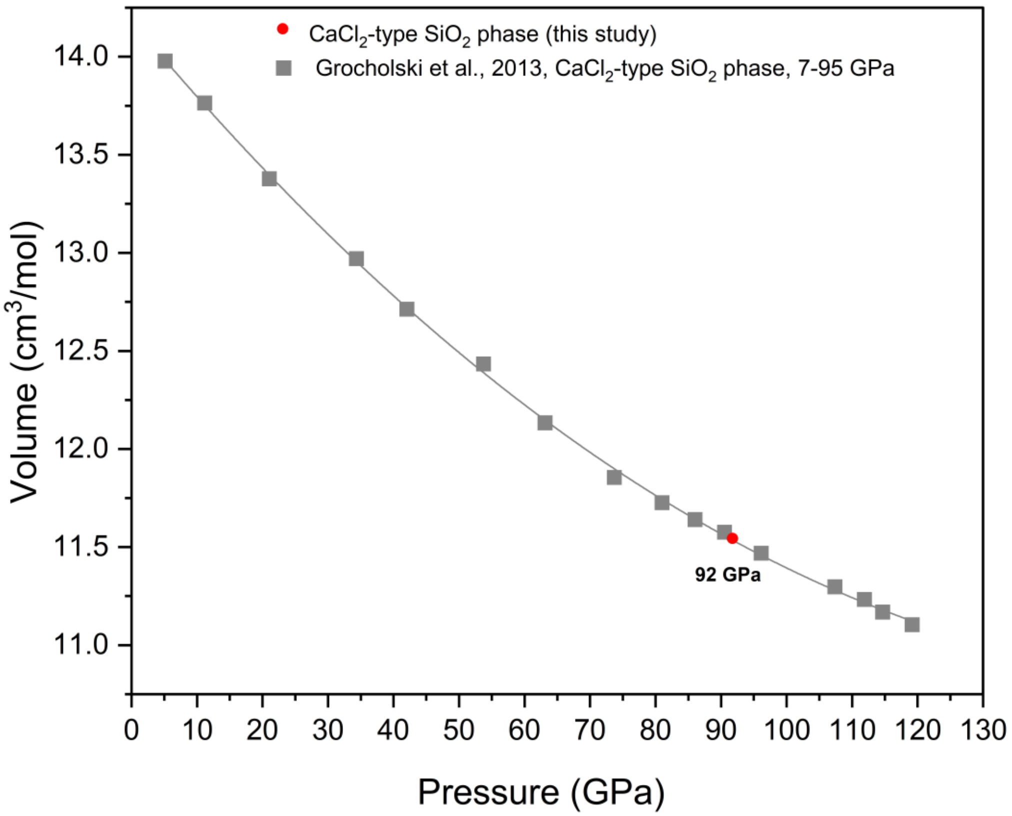

Fig. S10. Volume and pressure data of the high-pressure $CaCl_2$-type SiO_2 phase[13].

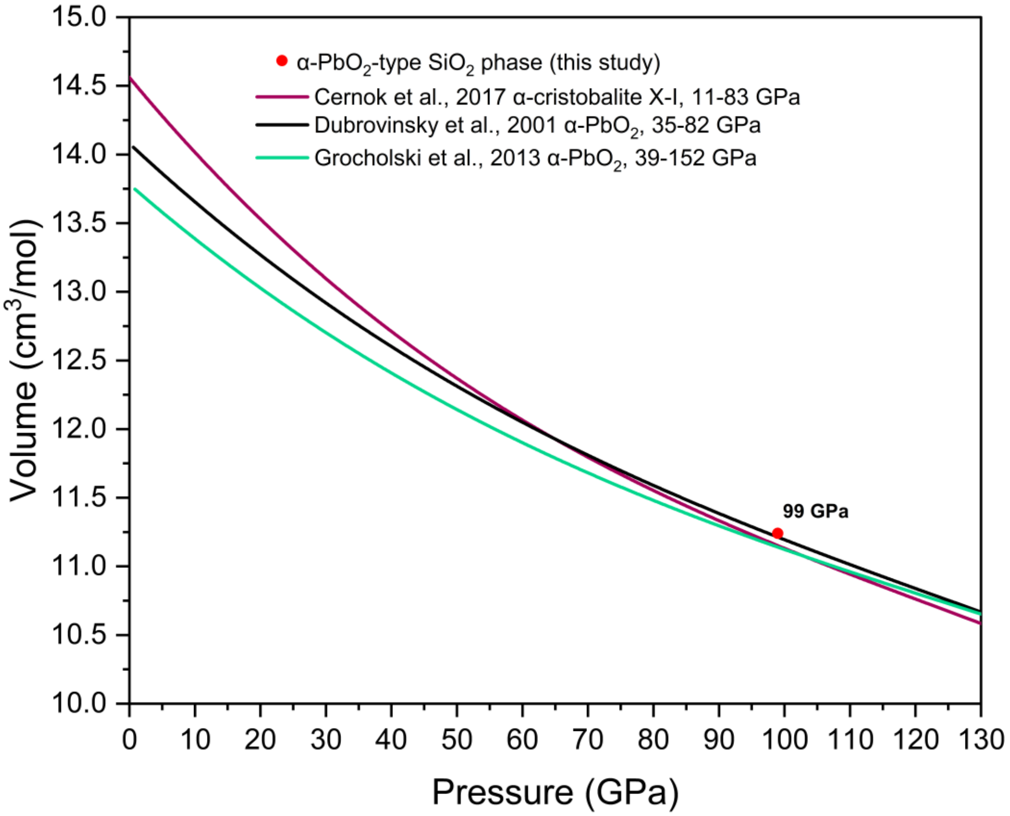

Fig. S11. Volume and pressure data of the high-pressure α-PbO_2-type SiO_2 phase, compared with previous studies[13–15].

Supplementary References

1. Popa, R. G., Tollan, P., Hermann, J. & Bachmann, O. Degassed versus pristine: Evaluating melt inclusions with a new ATR-FPA-FTIR calibration and water imaging method in rhyolitic melts. *Chem. Geol.* **615**, (2023).

2. Hammerli, J., Hermann, J., Tollan, P. & Naab, F. Measuring in situ CO_2 and H_2O in apatite via ATR-FTIR. *Contrib. to Mineral. Petrol.* **176**, 0–20 (2021).

3. Tollan, P., Ellis, B., Troch, J. & Neukampf, J. Assessing magmatic volatile equilibria through FTIR spectroscopy of unexposed melt inclusions and their host quartz: a new technique and application to the Mesa Falls Tuff, Yellowstone. *Contrib. to Mineral. Petrol.* **174**, 1–19 (2019).

4. Sandercock, 1970. GHOST v.7.0.0, The Table Stable Ltd.

5. Whitfield, C. H., Brody, E. M. & Bassett, W. A. Elastic moduli of NaCl by Brillouin scattering at high pressure in a diamond anvil cell. *Rev. Sci. Instrum.* **47**, 942–947 (1976).

6. Hanfland, M., Syassen, K., Fahy, S., Louie, S. G. & Cohen, M. L. Pressure dependence of the first-order Raman mode in diamond. *Phys. Rev. B* **31**, 6896–6899 (1985).

7. Akahama, Y. and & Kawamura, H. Pressure calibration of diamond anvil Raman gauge to 410 GPa. *J. Phys. Conf. Ser.* **215**, (2010).

8. Akahama, Y. and Kawamura, H. Pressure calibration of diamond anvil Raman gauge to 310 GPa. *J. Appl. Phys.* **100**, (2006).

9. Prescher, C. & Prakapenka, V. B. DIOPTAS: A program for reduction of two-dimensional X-ray diffraction data and data exploration. *High Press. Res.* **35**, 223–230 (2015).

10. Seto, Y. https://github.com/seto77/PDIndexer.

11. Murakami, M., Hirose, K., Ono, S. & Ohishi, Y. Stability of $CaCl_2$-type and α-PbO_2-type SiO_2 at high pressure and temperature determined by in-situ X-ray measurements. *Geophys. Res. Lett.* **30**, 1999–2002 (2003).

12. Andrault, D., Angel, R. J., Mosenfelder, J. L. & Le Bihan, T. Equation of state of stishovite to lower mantle pressures. *Am. Mineral.* **88**, 301–307 (2003).

13. Grocholski et al. Stability, metastability, and elastic properties of a dense silica polymorph, seifertite. *J. Geophys. Res. E Planets* **118**, 4745–4757 (2013).

14. Dubrovinsky *et al.* Pressure-induced transformations of cristobalite. *Chem. Phys. Lett.* **333**, 264–270 (2001).

15. Cernok, A. *et al.* Compressional pathways of α-cristobalite, structure of cristobalite X-I, and towards the understanding of seifertite formation. *Nat. Commun.* **8**, (2017).

16. WURM. https://www.wurm.info/index.php?id=2&view=detail&file=w000176.

17. Prokopenko, V. B., Dubrovinsky, L. S., Dmitriev, V. & Weber, H. P. In situ characterization of phase transitions in cristobalite under high pressure by Raman spectroscopy and X-ray diffraction. *J. Alloys Compd.* **327**, 87–95 (2001).

18. Goresy, A. E. l. D. *et al.* Seifertite, a dense orthorhombic polymorph of silica from the Martian meteorites Shergotty and Zagami. *Eur. J. Mineral.* **20**, 523–528 (2008).

19. Teter D. M. & Hemley R. J. High pressure polymorphism in silica. *Phys. Rev. Lett.* **80**, 2145–2148 (1998).

20. Dubrovinskaia *et al.* Direct transition from cristobalite to post-stishovite α-PbO_2-like silica

phase. *Eur. J. Mineral.* **13**, 479–483 (2001).

21. Dera, P., Prewitt, C. T., Boctor, N. Z. & Hemley, R. J. Characterization of a high-pressure phase of silica from the Martian meteorite Shergotty. *Am. Mineral.* **87**, 1018–1023 (2002).

22. Zhang *et al.* In-situ crystal structure determination of seifertite SiO_2 at 129 GPa: Studying a minor phase near Earth's core-mantle boundary. *Am. Mineral.* **101**, 231–234 (2016).

23. Hemley, R. J., Mao, H. K. & Chao, E. C. T. Raman spectrum of natural and synthetic stishovite. *Phys. Chem. Miner.* **13**, 285–290 (1986).

24. Cottaar S., Heister T., Rose I., Unterborn C., BurnMan: A lower mantle mineral physics toolkit. *Geochemistry Geophys. Geosystems* 1164–1179 (2014).

25. Myhill, R. *et al.* BurnMan – a Python toolkit for planetary geophysics , geochemistry and thermodynamics. *J. Open Source Softw.* **8**, 1–10 (2023).

26. Asahara, Y. *et al.* Acoustic velocity measurements for stishovite across the post-stishovite phase transition under deviatoric stress: Implications for the seismic features of subducting slabs in the mid-mantle. *Am. Mineral.* **98**, 2053–2062 (2013).

27. Stixrude, L. & Lithgow-Bertelloni, C. Thermodynamics of mantle minerals - II. Phase equilibria. *Geophys. J. Int.* **184**, 1180–1213 (2011).

28. Katsura, T. A Revised Adiabatic Temperature Profile for the Mantle. *J. Geophys. Res. Solid Earth* **127**, 1–11 (2022).

29. Murakami, M. & Takata, N. Absolute Primary Pressure Scale to 120 GPa: Toward a Pressure Benchmark for Earth's Lower Mantle. *J. Geophys. Res. Solid Earth* **124**, 6581–6588 (2019).

30. Dorfman, S. M., Prakapenka, V. B., Meng, Y. & Duffy, T. S. Intercomparison of pressure standards (Au, Pt, Mo, MgO, NaCl and Ne) to 2.5 Mbar. *J. Geophys. Res. Solid Earth* **117**, 1–15 (2012).

31. Sakai, T., Ohtani, E., Hirao, N. & Ohishi, Y. Equation of state of the NaCl-B2 phase up to 304 GPa. *J. Appl. Phys.* **109**, (2011).

32. Singh, A. K. & Balasingh, C. The lattice strains in a specimen (cubic system) compressed nonhydrostatically in an opposed anvil high pressure setup. *J. Appl. Phys.* **75**, 4956–4962 (1993).

33. Singh, A. K. & Kenichi, T. Measurement and analysis of nonhydrostatic lattice strain component in niobium to 145 GPa under various fluid pressure-transmitting media. *J. Appl. Phys.* **90**, 3269–3275 (2001).

34. Takemura, K. & Dewaele, A. Isothermal equation of state for gold with a He-pressure medium. *Phys. Rev. B - Condens. Matter Mater. Phys.* **78**, 1–13 (2008).

35. Liu, L., Bi, Y., Xu, J. & Chen, X. Ab initio study of the elastic properties of sodium chloride at high pressure. *Phys. B Condens. Matter* **405**, 2175–2180 (2010).

36. Tateno, S., Komabayashi, T., Hirose, K., Hirao, N. & Ohishi, Y. Static compression of B2 KCl to 230 GPa and its P-V-T equation of state. *Am. Mineral.* **104**, 718–723 (2019).

37. Shieh, S. R., Duffy, T. S. & Li, B. Strength and Elasticity of SiO_2 across the Stishovite–$CaCl_2$-type Structural Phase Boundary. *Phys. Rev. Lett.* **89**, 22–25 (2002).

38. Boehler, R. High-Pressure Experiments and the Phase Diagram of Lower Experiments and and the Phase Diagram. *Rev. Geophys.* **38**, 221–245 (2000).

39. Uts, I., Glazyrin, K. & Lee, K. K. M. Effect of laser annealing of pressure gradients in a

diamond-anvil cell using common solid pressure media. *Rev. Sci. Instrum.* **84**, (2013).

40. Smith *et al.* A CO_2 laser heating system for in situ high pressure-temperature experiments at HPCAT. *Rev. Sci. Instrum.* **89**, (2018).

41. Lin, Y. *et al.* Hydrous SiO_2 in subducted oceanic crust and H_2O transport to the core-mantle boundary. *Earth Planet. Sci. Lett.* **594**, (2022).

42. Ballmer M. D., Schumacher L., Lekic V., Thomas C., Ito G. Compositional layering within the large low shear-wave velocity provinces in the lower mantle. *Geochemistry Geophys. Geosystems* **17**, 1312–1338 (2016).

43. Shieh, S. R., Duffy, T. S. & Shen, G. X-ray diffraction study of phase stability in SiO_2 at deep mantle conditions. *Earth Planet. Sci. Lett.* **235**, 273–282 (2005).

44. Sun, N., Shi, W., Mao, Z., Zhou, C. & Prakapenka, V. B. High Pressure-Temperature Study on the Thermal Equations of State of Seifertite and $CaCl_2$-Type SiO_2. *J. Geophys. Res. Solid Earth* **124**, 12620–12630 (2019).

45. Brown, J. M. & Shankland, T. J. Thermodynamic parameters in the Earth as determined from seismic profiles. *Geophys. J. R. Astron. Soc.* **66**, 579–596 (1981).

46. Wang, W. *et al.* Velocity and density characteristics of subducted oceanic crust and the origin of lower-mantle heterogeneities. *Nat. Commun.* **11**, 1–8 (2020).

47. Hirose, K., Takafuji, N., Sata, N. & Ohishi, Y. Phase transition and density of subducted MORB crust in the lower mantle. *Earth Planet. Sci. Lett.* **237**, 239–251 (2005).

48. Gréaux, S. *et al.* Sound velocity of $CaSiO_3$ perovskite suggests the presence of basaltic crust in the Earth's lower mantle. *Nature* **565**, 218–221 (2019).

49. Thomson, A. R. *et al.* Seismic velocities of $CaSiO_3$ perovskite can explain LLSVPs in Earth's lower mantle. *Nature* **572**, 643–647 (2019).

50. Murakami, M., Sinogeikin, S. V., Hellwig, H., Bass, J. D. & Li, J. Sound velocity of $MgSiO_3$ perovskite to Mbar pressure. *Earth Planet. Sci. Lett.* **256**, 47–54 (2007).

51. Criniti, G., Kurnosov, A., Boffa Ballaran, T. & Frost, D. J. Single-Crystal Elasticity of $MgSiO_3$ Bridgmanite to Mid-Lower Mantle Pressure. *J. Geophys. Res. Solid Earth* **126**, (2021).

52. Dorfman, S. M. & Duffy, T. S. Effect of Fe-enrichment on seismic properties of perovskite and post-perovskite in the deep lower mantle. *Geophys. J. Int.* **197**, 910–919 (2014).

53. Mashino, I., Murakami, M., Miyajima, N. & Petitgirard, S. Experimental evidence for silica-enriched Earth's lower mantle with ferrous iron dominant bridgmanite. *Proc. Natl. Acad. Sci. U. S. A.* **117**, 27899–27905 (2020).

54. Murakami, M., Ohishi, Y., Hirao, N. & Hirose, K. A perovskitic lower mantle inferred from high-pressure, high-temperature sound velocity data. *Nature* **485**, 90–94 (2012).

55. Jackson, J. M., Zhang, J., Shu, J., Sinogeikin, S. V. & Bass, J. D. High-pressure sound velocities and elasticity of aluminous $MgSiO_3$ perovskite to 45 GPa: Implications for lateral heterogeneity in Earth's lower mantle. *Geophys. Res. Lett.* **32**, 1–4 (2005).

56. Ohta, K., Hirose, K., Shimizu, K., Lay, T. & Cruz, S. Phase transitions in pyrolitic mantle and MORB materials and their electrical conductivities at lowermost mantle conditions. *Earth Planet. Sci. Lett.* **267**, 107–117 (2008).
